# Novel pathogenic splicing mutation in *COL11A1* in a patient with Stickler syndrome verified by minigene splicing assay

**DOI:** 10.3389/fgene.2025.1642604

**Published:** 2025-08-22

**Authors:** Jie Zhang, Yaxin Huang, Yafei Deng, Xiaochun Yang, Hong Shi, Yuecheng Yang, Tao Lv, Yuanlong Yan, Ming He, Fang Liu

**Affiliations:** ^1^ Medical School, Kunming University of Science and Technology, The First People’s Hospital of Yunnan Province, Kunming, Yunnan, China; ^2^ Department of Medical Genetics, The First People’s Hospital of Yunnan Province, Kunming, China; ^3^ National Health Commission Key Laboratory of Preconception Health Birth in Western China, The First People’s Hospital of Yunnan Province, Kunming, China; ^4^ Department of Ophthalmology, The First People’s Hospital of Yunnan Province, Kunming, China; ^5^ State Key Laboratory of Primate Biomedical Research, Institute of Primate Translational Medicine, Kunming University of Science and Technology, Kunming, Yunnan, China; ^6^ The Second Affiliated Hospital of Kunming Medical University, Kunming, Yunnan, China; ^7^ Department of Biochemistry and Molecular Biology, Faculty of Basic Medical Science, Kunming Medical University, Kunming, Yunnan, China

**Keywords:** *de novo* novel splicing variant, whole-exome sequencing, minigene splicing assay, *COL11A1* gene, Stickler syndrome

## Abstract

**Background:**

Stickler syndrome (STL) is a group of related connective tissue disorders characterized by heterogeneous clinical presentations with varying degrees of orofacial, ocular, skeletal, and auditory abnormalities. However, this condition is difficult to diagnose on the basis of clinical features because of phenotypic variability. Thus, expanding the variant spectrum of this disease will aid in achieving a firm definitive diagnosis of STL.

**Methods:**

Comprehensive examinations, including ophthalmology, otology, and orthopedic evaluations, were performed to identify the disease phenotype of the proband. Furthermore, whole-exome sequencing (WES) and Sanger sequencing were performed to identify the molecular basis of the disease. *In silico* analysis and a minigene splicing assay were conducted to verify the pathogenicity of the splice site variant. The clinical phenotypes of the reported STL patients were then reviewed.

**Results:**

The proband presented mild symptoms with early-onset high myopia and mild scoliosis. A novel *de novo* splicing variant (NM_080629.3: c.4069-1G>T), in the *COL11A1* gene was identified in the proband via WES and confirmed via Sanger sequencing. Minigene splicing assays verified that this variant resulted in abnormal splicing of the *COL11A1* transcripts because of the skipping of exon 54 and retention of 21 bp in intron 53. The literature review revealed that the most common phenotypes associated with STL type 2 include myopia and hearing impairment.

**Conclusion:**

We identified a novel acceptor splice site variant causing aberrant splicing of *COL11A1*. Our findings expand the variant spectrum of this gene and provide a precise genetic diagnosis of STL that could be helpful in genetic counseling, reproductive prevention, and treatment of long-term complications of this disorder.

## 1 Introduction

Stickler syndrome (STL) was first reported in 1965 by Gunnar Stickler. It includes a group of related connective tissue disorders with heterogeneous clinical presentations with varying degrees of orofacial, ocular, skeletal, and auditory abnormalities. The incidence of STL among neonates is estimated to be approximately 1 in 7,500–9,000 neonates. There are two inheritance patterns of STL: autosomal dominant inheritance and recessive inheritance. STL caused by pathogenic variants in *COL2A1*, *COL11A1*, or *COL11A2* is inherited in an autosomal dominant manner (Rose et al., 2005; Snead et al., 2020). In contrast, STL caused by pathogenic variants in *COL9A1*, *COL9A2*, and *COL9A3* has been reported to be inherited in a recessive manner (Nixon et al., 2022; Van Camp et al., 2006). There are six types of STL according to the different pathogenic genes. Among these, three types that are inherited via an autosomal dominant pattern are more commonly observed. STL type 1 (OMIM#108300), which is caused by mutations in the *COL2A1* gene, accounts for approximately 80.0%–90.0% of all STL cases. STL type 2 (OMIM#604841) is caused by mutations in the *COL11A1* gene; it accounts for 10.0%–20.0% of all STL cases. The remaining *COL11A2*-related STL type 3 (OMIM#184840) is also referred to as otospondylomegaepiphyseal dysplasia (OSMED) (Boothe et al., 2020; Soh et al., 2022). The recessive STL types are uncommon and include types 4, 5, and 6, which are caused by biallelic mutations in the *COL9A1*, *COL9A2*, and *COL9A3* genes, respectively (Nixon et al., 2022).

The *COL11A1* gene is mapped to human chromosome 1p21.1, spanning 232.05 kp of genomic DNA, and comprises 67 exons. The *COL11A1* gene encodes the α1 chain of type XI collagen fibers. Type XI collagen is a minor fibrillar collagen expressed in the cartilage, vitreous humor, intervertebral discs, and inner ear. It is a heterotrimer composed of α1, α2, and α3 chains (Morris and Bächinger, 1987). Type XI collagen is usually co-expressed with type II collagen and regulates the fibril diameter of type II collagen (Blaschke et al., 2000; Richards and Snead, 2022).

STL is a multisystem disorder with significant genetic and phenotypic heterogeneity. Accordingly, this condition may be difficult to diagnose on the basis of clinical features. With the use of next-generation sequencing (NGS) in laboratories, the diagnosis can be confirmed by molecular genetic analysis. Herein, a novel splice site variant, *COL11A1*: NM_080629.3: c.4069-1G>T, was identified in a 6-year-old girl through whole-exome sequencing (WES). Furthermore, we conducted minigene splicing assays to identify a previously unknown *COL11A1* splice variant in a Chinese family with STL.

## 2 Materials and methods

### 2.1 Ethical approval

This study was approved by the Ethical Review Board of the First People’s Hospital of Yunnan Province. Informed consent was obtained from all participants and the participants’ legal guardians before the collection of clinical data and genomic samples (Approval No: KHLL2025-KY026).

### 2.2 Clinical assessment of the proband

The proband visited our outpatient clinic because of high myopia, and her parents were eager to prevent the recurrence of the same situation in the offspring. Information regarding the family history was obtained through interviews with the patient’s parents. A detailed physical examination and developmental assessment were performed. The proband was subsequently tested via refractive error measurement, best-corrected visual acuity (BCVA), slit lamp examination, and fundus photography. Moreover, audiometry and spine X-ray examinations were performed.

### 2.3 WES and sanger sequencing

Genomic DNA was extracted from the peripheral venous blood of the patient and her parents. WES was performed as previously described (Mamanova et al., 2010). Firstly, the DNA was interrupted and the library was prepared, and then the DNA of the target gene exon and adjacent cut region was captured and enriched by Roche KAPA HyperExome chip (KAPA Biosystems, Boston, MA, United States of America). Finally, the DNA was screened using NGS assay based on the DNA sequencer of MGISEQ-2000 (BGI, China). The average depth of sequencing in the target region was ≥180X, and the proportion of sites with average depth >20X in the target region was >95%. The sequencing fragments were compared with the human reference genome (UCSC hg19) by BWA to remove duplicates. GATK was used for base mass value correction for SNV, INDEL and genotyping. Exon level copy number variation was detected using ExomeDepth. Variants were annotated and screened based on clinical information, population databases, disease databases, and biological information prediction tools. The pathogenicity of the variants was determined according to the ACMG guidelines.

The candidate *COL11A1* variant was validated by Sanger sequencing for the patient and her parents. Primers were designed for the variant of the splice site variant in intron 53 of the *COL11A1* gene. The forward (AAG​CTA​GCA​CTG​GAC​TTT​TGA​C) and reverse primers (AGG​TAT​CTG​AAA​TAG​GGC​TGA​G) were used for amplification. The PCR products were further subjected to Sanger sequencing.

### 2.4 *In silico* variant analysis

Four *in silico* splice site prediction programs, MaxEntScan (https://www.genes.mit.edu/burgelab/maxent/Xmaxentscan_scoreseq.html), NNSPLICE 0.9 (https://www.fruitfly.org/seq_tools/splice.html), NetGene2 (https://services.healthtech.dtu.dk/services/NetGene2-2.42), and alternative splice site predictor (http://wangcomputing.com/assp), were used to predict the splice variants to evaluate variant pathogenicity. The protein structure models of AlphaFold (https://alphafold.ebi.ac.uk/entry/P12107) and SWISS-MODEL (https://swissmodel.expasy.org/) were used to explore the effect of pathogenic variant in the *COL11A1* gene on protein structure. Subsequently, the three-dimensional structure of the protein models was analyzed and visualized using PyMOL. The prediction method was carried out using standard procedures.

### 2.5 Minigene assay

Using genomic DNA from the proband and his parents as a template, primers were designed to amplify the *COL11A1* gene, including the exon 53, intron 53, exon 54, part of intron 54, and exon 55 regions. Primers were designed via seamless cloning to amplify heterozygous genomic DNA carrying the c.4069-1G>T variant site to obtain two target insertion gene fragments, *COL11A1-A* and *COL11A1-B*. The sequences of primers used were as follows: AF: AAG​CTT​GGT​ACC​GAG​CTC​GGA​TCC​GGT​CAA​GAT​GGT​GTT GGT​GGT​GAC​AAG​G; AR: CCT​GAG​TGT​GGA​GTT​AAT​GTT​CAG​GAT​ACC​ACC​AAA​GTC; BF: CAT​TAA​CTC​CAC​ACT​CAG​GGT​GGC​GAT​TTT​AAT​TC; and BR: TTA​AAC​GGG​CCC​TCT​AGA​CTC​GAG​CTT​AGC​ACC​TTT​TTC​ACC​TTG​TCT​TCC​CTC​T.

The vector pMini-CopGFP0 was digested via the *BamH* I/*Xho* I double endonuclease, and the digested products and the *COL11A1-A* fragment and *COL11A1-B* fragment were recombined. The correct wild type (WT) and mutant type (MT) minigene plasmids were selected, and 293T cells were subsequently transfected with the WT and MT plasmids via Lipofectamine 3000 according to the instructions. After transfection into 293T cells for 48 h, the transcripts were analyzed via RT‒PCR. The primers used for RT‒PCR amplification were as follows: primer sequence F: GGC​TAA​CTA​GAG​AAC​CCA​CTG​CTT​A; R: CTT​AGC​ACC​TTT​TTC​ACC​TTG​TCT​TC. The PCR products for both the WT and MT variants were subjected to 1.0% agarose gel electrophoresis, followed by Sanger sequencing.

### 2.6 Review of the *COL11A1* genotype and phenotypic data

We summarized the phenotypes and compiled the incidence of different phenotypes of STL from a previous review (Boothe et al., 2020), systematic review (Boysen et al., 2020; Frederic et al., 2012), and large-scale studies (Alexander et al., 2020; Bath et al., 2021; Rose et al., 2001; Zimmermann et al., 2019). To comprehensively identify previously published patients with *COL11A1* splice site alteration variants, we used ClinVar to identify pathogenic and likely pathogenic *COL11A1* variants.

## 3 Results

### 3.1 Case presentation

**FIGURE 1:**
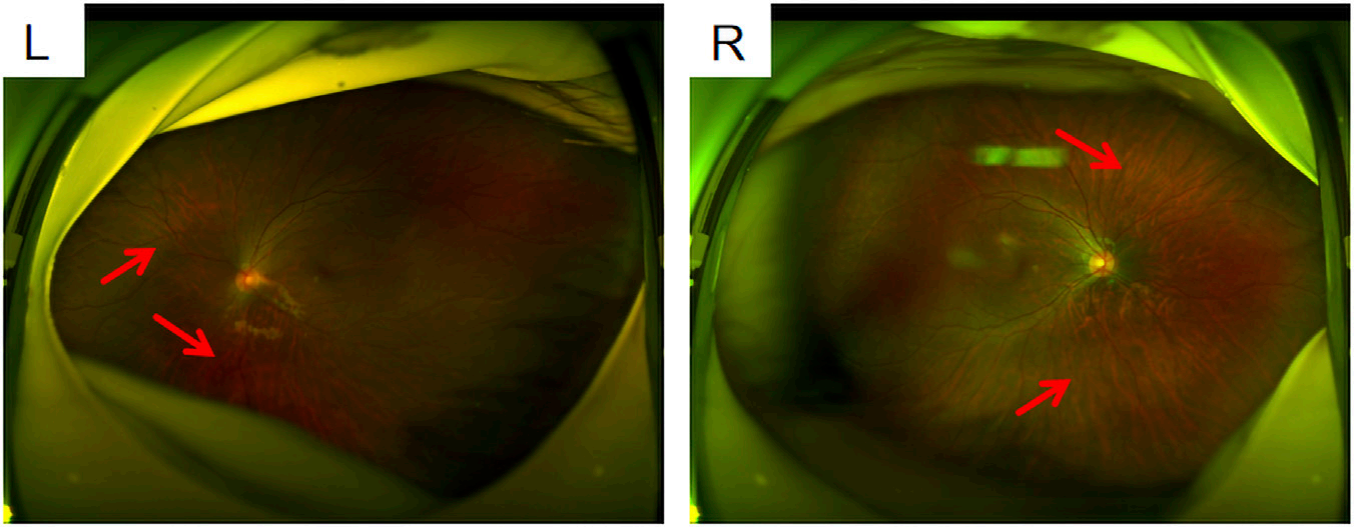
Fundus photographs L: left eye; R: right eye; both eyes show a leopard fundus. It was more obvious in the right eye (where the red arrow is pointed). Spot-like changes resembling leopards appear on the retina of the fundus.

**FIGURE 2:**
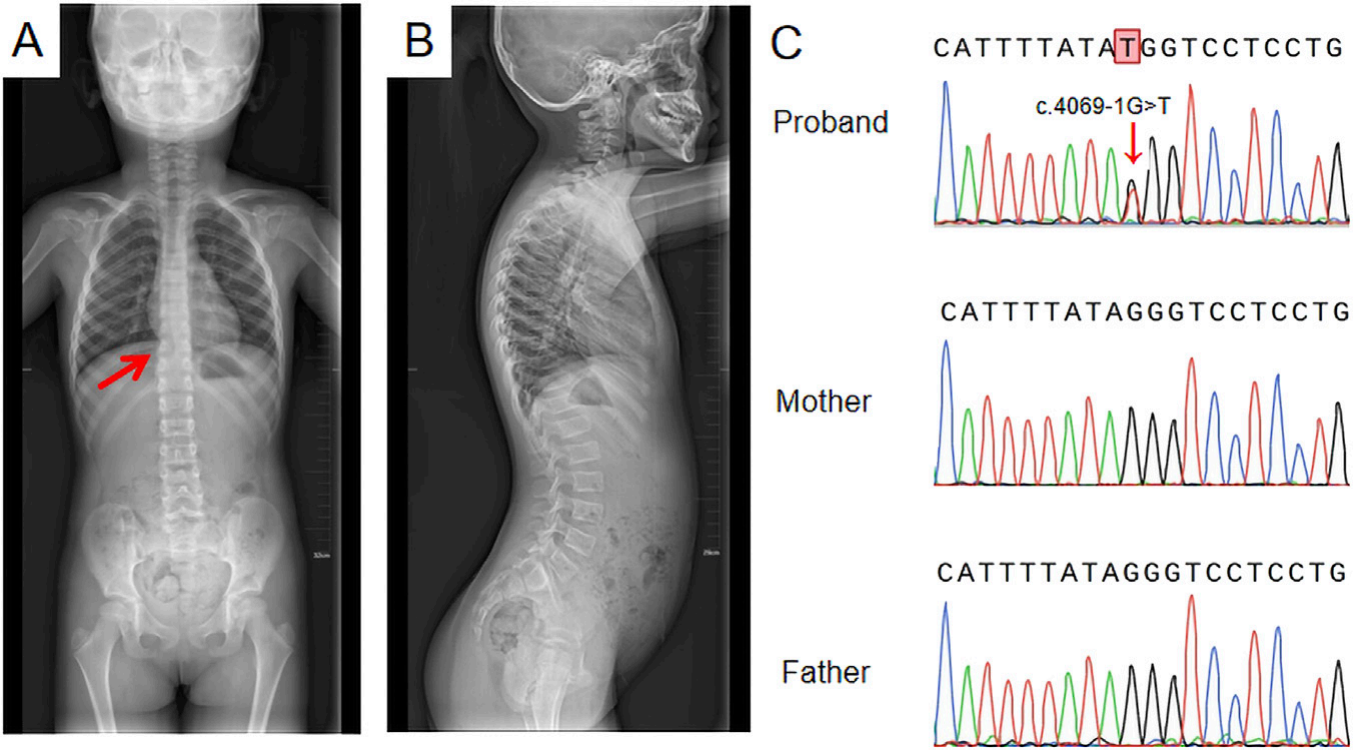
Spinal X-ray and Sanger sequencing results **(A)** Full-length anteroposterior radiograph of the spine; the red arrow indicates mild scoliosis; **(B)** Full-length lateral radiograph of the spine showing no significant change; **(C)** Sanger sequencing results: a *de novo* heterozygous splice site variant, c.4069-1G>T, was identified.

The proband, a 6-year-old girl, was the first child of nonconsanguineous parents. No family history and medical history was observed within her family. Her mother had a normal pregnancy, and a cesarean section was performed at term. The patient was found to have high myopia at the age of 3 years. Ophthalmologic assessment confirmed bilateral highly myopic astigmatism in the right eye (RE): −8.00/–1.00 × 180 and left eye (LE): −5.50/–1.00 × 35. Slit lamp examination revealed no abnormalities in the conjunctiva, cornea, or lens. Fundus examination revealed leopard fundus (Figure 1). The results of the spinal X-ray examination revealed mild scoliosis (Figures 2A,B). Her height was 114 cm, which was below the 10th percentile. Her motor and mental development were normal. No clinical characteristics of orofacial abnormalities were detected. Furthermore, audiometric examination revealed no significant abnormalities.

### 3.2 WES and sanger sequencing

A *de novo* heterozygous splice site variant, c.4069-1G>T, in the *COL11A1* gene was identified via WES. This variant was confirmed to not be previously annotated in ESP databases, the 1,000 Genomes Project, the ExAC Browser, or the gnomAD database. Sanger sequencing of the *COL11A1* gene confirmed the c.4069-1G>T heterozygous variant in the patient, and neither of her parents carried the variant (Figure 2C). According to the ACMG guidelines for variant pathogenicity, c.4069-1G>T was classified as “likely pathogenic” (PVS1_Moderate + PS2_Moderate + PM2).

### 3.3 *In silico* variant analysis

**FIGURE 3:**
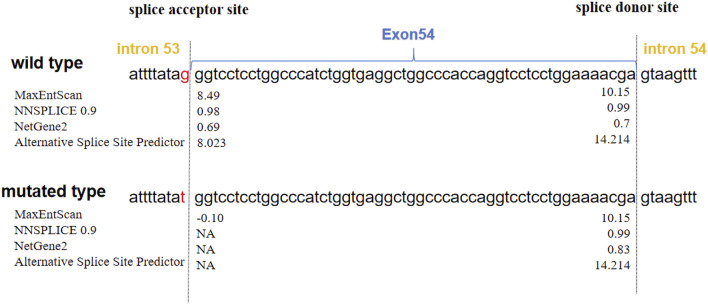
Predictions of the scores of acceptor and donor splice sites of *COL11A*1 exon 54. The blue closed arrow indicates exon 54. The splice sites are located on either side of the dashed line. The scores calculated via four splice site prediction programs are displayed next to each splice site. MaxEntScan and Alternative splice site predictor scores: higher positive values indicate stronger splice sites. NNSPLICE and NetGene2 scores range from 0.0 to 1.0, with higher scores indicating higher confidence in a true splice site. The scores significantly decreased, and the mutated type showed a loss of the acceptor splice site, indicating that the variant weakened the acceptor splice site. NA: no splicing sites.

The prediction scores for acceptor and donor splice sites in the wild type and mutated type are shown in Figure 3. A higher score predicts a strong splice site. The scores of the acceptor splice site of intron 53 were markedly decreased from 8.49 to −0.10 for the c.4069-1G>T variant determined via MaxEntScan. The other three prediction programs showed that the acceptor splice site mutated from G to T resulted in the disappearance of the acceptor splice site. These analyses revealed that the variant weakened the acceptor splice site.

### 3.4 Minigene assay

**FIGURE 4:**
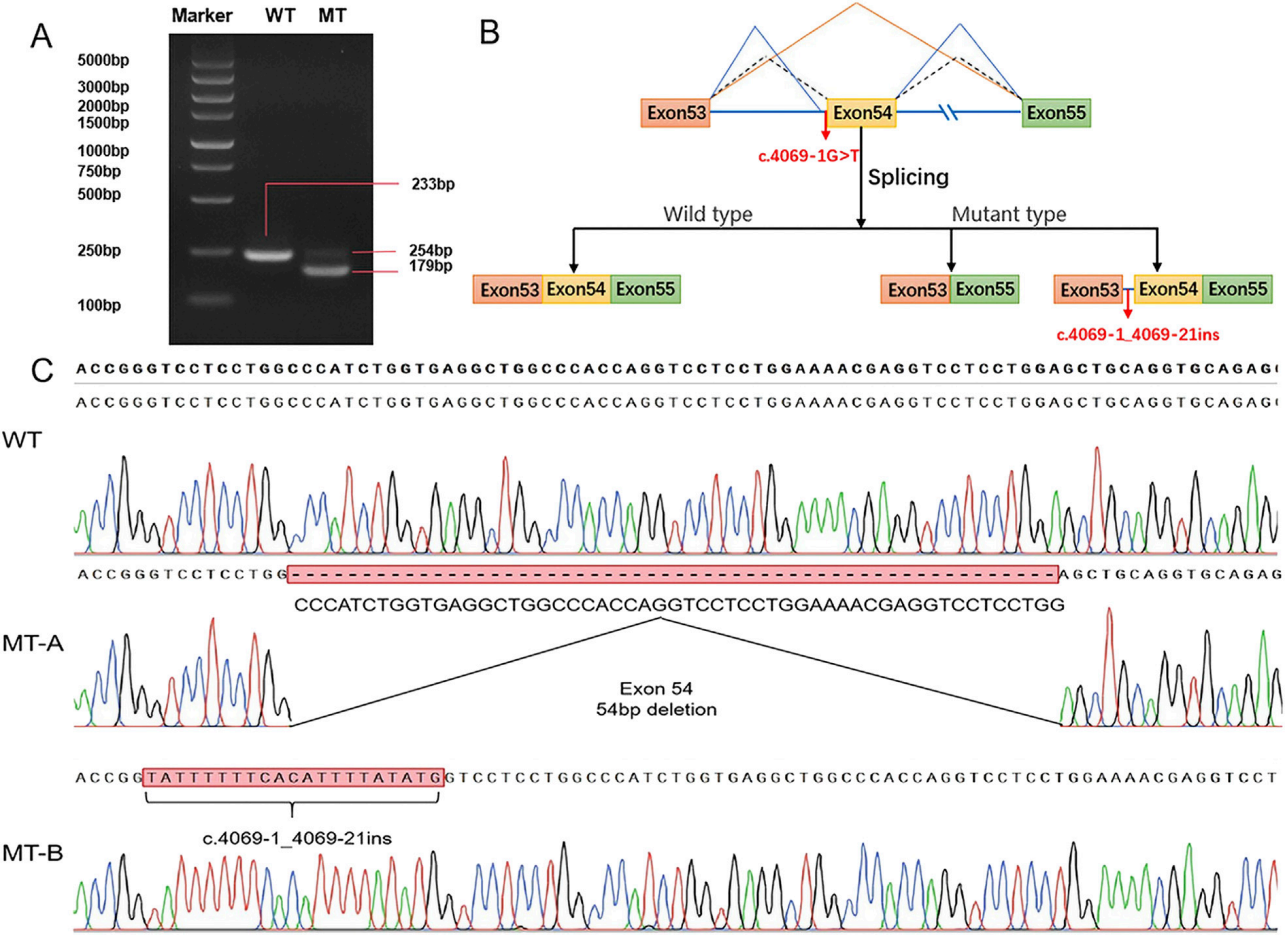
Minigene vector constructs for the *in vitro* splicing assay **(A)** Electrophoretogram of RT‒PCR products: Lane 1: marker; Lane 2: wild type (WT) plasmid: 233 bp product; Lane 3: mutant type (MT) plasmid MT-A product (179 bp), MT-B product (254 bp). **(B)** Schematic representation of the splicing events associated with this *COL11A1* variant. **(C)** Sanger sequencing of RT‒PCR products: variant c.4069-1G>T resulted in a 54 bp deletion caused by exon 54 skipping and retention of 21 bp in intron 53.

To determine the splicing pattern and verify the pathogenicity of the splice site variant (Figure 4B), we constructed a minigene vector via an *in vitro* splicing assay for both the WT and MT *COL11A1* gene sequences from exons 53 to 55. RT‒PCR revealed that the cells containing the WT plasmid produced an amplicon of 233 bp. In contrast, the cells transfected with the c.4069-1G>T variant sequence produced two fragments, a 254 bp band and a 179 bp band (Figure 4A). Subsequently, Sanger sequencing revealed that the two mutant fragments presented exon 54 skipping and a retention of 21 bp in intron 53 (Figure 4C). These findings suggest that the variant in the *COL11A1* gene produced two aberrantly spliced cDNA.

### 3.5 Three-dimensional structure of the protein

**FIGURE 5:**
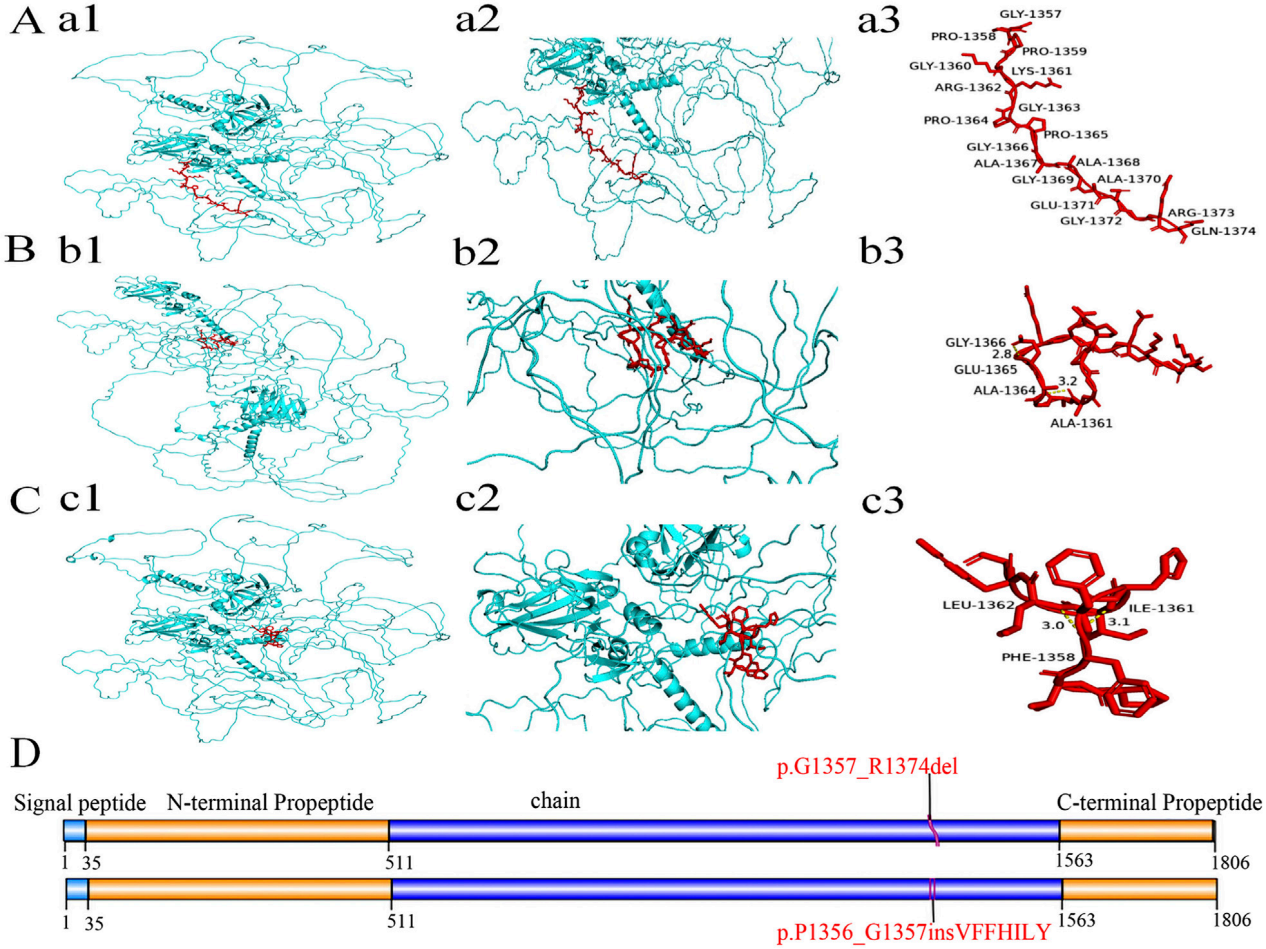
The domain and three-dimensional structures of the wild type and variant COL11A1 proteins. **(A‐C)** Protein structure analysis including diagrams of three proteins, with each displaying two views (one zoomed) and annotated red amino acid sequences. The 3D structure of COL11A1 wild type (a1) and two variant proteins (b1, c1). Local magnification of the deleted and inserted areas in COL11A1 proteins (a2: WT, b2: p.G1357_R1374del, c2: p.P1356_G1357insVFFHILY). In the 18-amino acid region of the wild type (a3), no hydrogen bond interaction was found. But both variant proteins (b3, c3) have generated new hydrogen bonds in the mutant regions (b3: Gly1366 and Glu1365, Ala1364 and Ala1361; c3: Leu1362 and Phe1358, Ile1361 and Phe1358). These changes may lead to the instability of local peptide chainstructure or cause abnormal folding of the helix. **(D)** Schematic representation of COL11A1 protein domains. The red font indicates deletion of 18 amino acids and insertion of seven amino acids. In accordance with HGVS recommendations, amino acid changes are described using single-letter abbreviations (G: Gly; R: Arg; P: Pro; V: Val; F: Phe; H: His; I: Ile; L: Leu; Y: Tyr).

The three-dimensional (3D) structure prediction of the wild and mutant models of the COL11A1 protein were shown in Figure 5. Two aberrantly spliced cDNA resulted in one protein variant with a deletion of 18 native amino acids (p.Gly1357_Arg1374del) (Figures 5b1,b2,b3), and another variant with an insertion of seven amino acids (p.Pro1356_Gly1357insValPhePheHisIleLeuTyr) (Figures 5c1,c2,c3). Compared with the wild type sequence, both variant proteins had altered amino acid sequences. Further structural analysis suggested that both mutant proteins form new hydrogen bonds in the mutated regions, leading to abnormal helix folding.

### 3.6 Review of the *COL11A1* genotype and phenotypic data

Owing to the high phenotypic heterogeneity of STL, we summarized the clinical phenotypes of partially reported STL cases. As shown in Table 1, the most common phenotypes included myopia (88%) (Boysen et al., 2020) and hearing impairment (82.5%) (Frederic et al., 2012) in STL type 2. In our patient, myopia was the main presentation, but hearing abnormality was not observed.

**TABLE 1:** Comparison of the phenotypes of Stickler syndrome reported in previous studies and this study.

Genotype and Phenotype Information	Present case	Previous studies
Genotype		
Gene	*COL11A1*	*COL11A1*

Mutation	c.4069-1G>T	c.4069-1G>C	Others
Phenotype			
Ocular			
Myopia	+	+	88% (30/34)
Cataract	−	−	59% (20/34)
Vitreous phenotype	−	+	Beaded
Glaucoma	−	NA	No information
Retinal detachment	−	−	38% (13/34)
Orofacial			
Pierre–Robin sequence	−	−	1.5% (1/65)
Bifid uvula	−	−	1.5% (1/65)
High-arched palate	−	−	1.5% (1/65)
Micrognathia	−	+	No information
Midfacial hypoplasia	−	+	No information
Cleft palate (Previous cleft repair)	−	+	24.6% (16/65)
Hearing impairment	−	+	82.5% (33/40)
Skeletal			
Height	114 cm (p10)	96.5 cm (mean)	No information
Spondyloepiphyseal dysplasia	−	−	0% (0/7)
Scoliosis	+	−	1.5% (1/65)
Joint hypermobility	−	NA	33.8% (22/65)
Others*	−	NA	No information

Note: p10: the 10th percentile; NA: not assessed; *: osteoarthritis before the age of 40 years, hyper-extensibility, talipes equinovarus, pectus carinatum, and pectus.

Other common clinical phenotypes of STL type 2 included cataract (59%), retinal detachment (38%), cleft palate (24.6%), and joint hypermobility (33.8%). Other relatively rare symptoms, such as Pierre-Robin sequence (1.5%), Bifid uvula (1.5%), high-arched palate myasthenia (1.5%), and scoliosis (1.5%), were reported in less than 10% of STL type 2 cases (Zimmermann et al., 2019). Among the rare symptoms, our patient presented with mild scoliosis.

**FIGURE 6:**
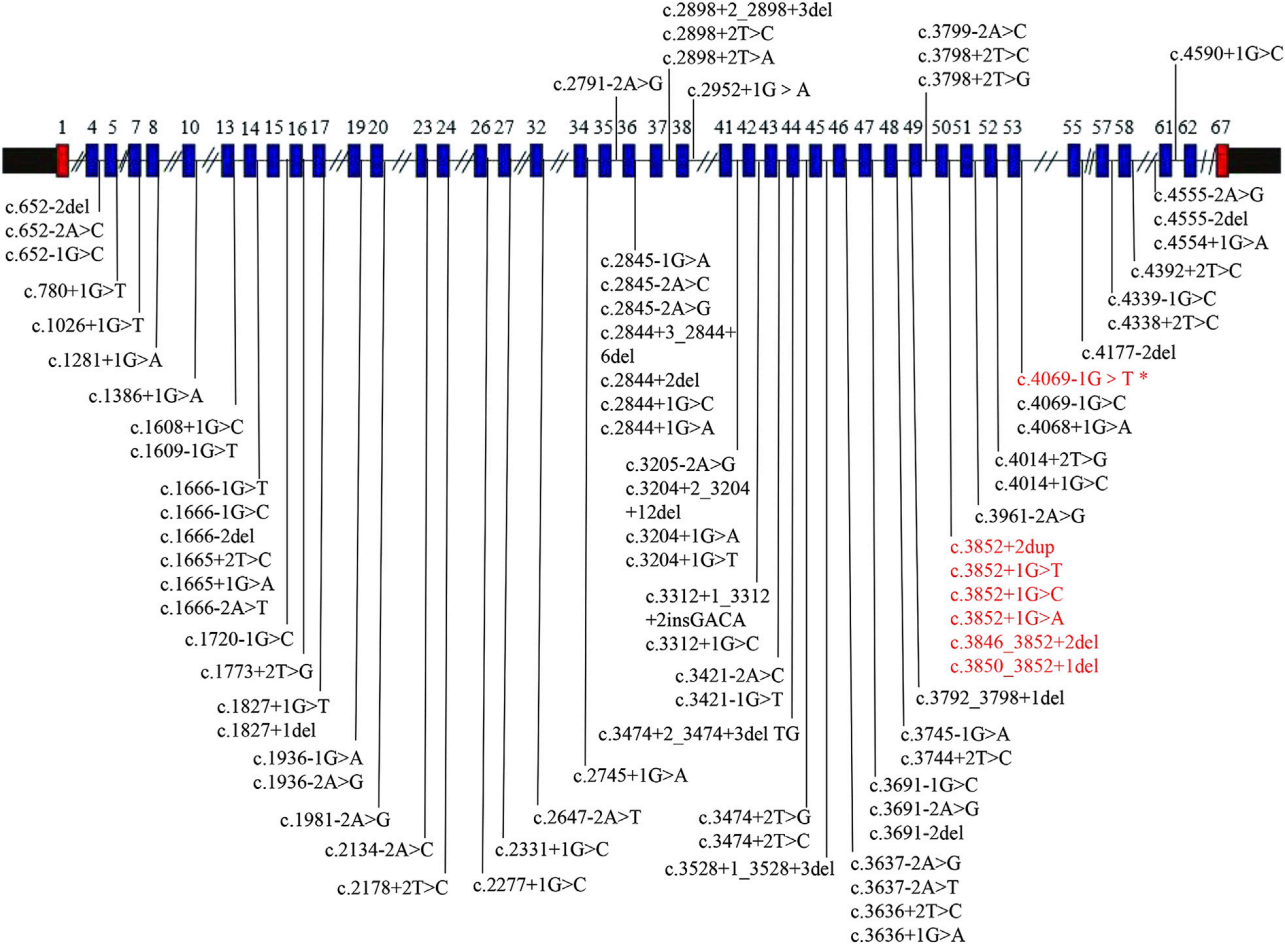
*COL11A1* gene schematic representation reporting pathogenic and likely pathogenic splicing variants. All variants were collected from ClinVar (https://www.ncbi.nlm.nih.gov/clinvar), accessed at 27 October 2024. The blue box shows the exons with splice site variants. The text colored red represents the hotspot variant region, and the text colored red (*) represents the variant in our case.

ClinVar has reported 186 pathogenic and likely pathogenic *COL11A1* variants, with a total of 84 sites with splicing variants (Figure 6). For the sites with splicing variants, most variants affect +1 or +2 residues at the 5′donor splice site and −1 or −2 residues at the 3′acceptor splice site.

## 4 Discussion

STL is a multisystem disorder with significant genetic and phenotypic heterogeneity. Heterozygous variants in the *COL11A1* gene usually cause type 2 STL. The biallelic variant in *COL11A1* is typically associated with severe lethal fibrochondrogenesis (Tompson et al., 2010). Here, we identified a *de novo* heterozygous splice site variant that caused STL type 2 in an autosomal dominant manner.

In the present study, the proband presented high myopia at the age of 3 years. Ophthalmologic assessment confirmed bilateral highly myopic astigmatism. Fundus examination revealed a leopard fundus. The results of the spinal X-ray revealed mild scoliosis. The patient’s height was 114 cm, which was below the 10th percentile. This condition is difficult to clearly diagnose on the basis of these nonspecific phenotypes. We applied WES to identify possible causative gene variants, revealing a *de novo* heterozygous splice site variant, c.4069-1G>T, located at the exon-intron boundary of the *COL11A1* gene in the proband.

To verify the pathogenicity of the splice site variant, we used four splice site prediction programs. Bioinformatics analysis revealed that the variant weakened the acceptor splice site and may cause exon 54 to be skipped or retained by other splice sites. In most cases (98.7%), the exon‒intron boundary has a highly conserved sequence that serves as a splicing recognition signal, containing classical splice sites of GT-AG at the 5′and 3′ends of the intron. Most classical variants affect +1 or +2 residues at the 5′donor splice site and −1 or −2 residues at the 3′acceptor splice site (Anna and Monika, 2018; Montes et al., 2019). In our study, this *de novo* variant was located exactly at the −1 residue of the 3′acceptor splice site, so we highly suspected that it was pathogenic.

We constructed a minigene vector to deeply analyse the splice site variant. Our experiments revealed that this variant affected the normal splicing of RNA by skipping exon 54 and inserting 21 bp, which produced two aberrantly spliced cDNA. Meanwhile, we further evaluated the effect of the pathogenic variant in the *COL11A1* gene on the protein structure. We found that the variant proteins were predicted to have altered hydrogen bonding and abnormal protein lengths. These hydrogen bond alterations may reduce protein stability or cause abnormal folding of the helix. Additionally, changes in the protein’s length may lead to mismatches with other normal-length chains, ultimately affecting the formation of the collagen triple helix. Previous studies have shown that pathogenic variants in *COL11A1* generally exert a dominant-negative effect on type XI collagen heterotrimer formation (Spranger, 1998). *COL11A1* encodes the α1 chain of collagen XI, which is composed of three α chains. Mutant chains combine with normal chains will generate abnormal triple helices, leading to collagen structure abnormalities (Prockop and Kivirikko, 1995; Myllyharju, 2004; Myllyharju and Kivirikko, 2009). In our study, we revealed that this *de novo* variant affected the normal splicing of RNA, and two abnormal splicing products were generated. We infer that aberrantly translated proteins have a negative impact on the collagen triple helix structure, ultimately leading to a disease phenotype.

Heterozygous pathogenic *COL11A1* variants are predominantly splice site alterations and missense variants. Furthermore, ClinVar has reported 186 pathogenic and likely pathogenic *COL11A1* variants, with a total of 84 sites with splicing variants. Intron 50 is a variant hot spot (Acke et al., 2014; Majava et al., 2007). However, c.4069-1G>T variant found in this study was located at intron 53. Interestingly, the similar variant c.4069-1G>C in the same splice site has been previously reported (Majava et al., 2007). A comparison of cases from the literature revealed variability in disease phenotypes (Table 1 shows a summary of these two variants). In the study of Marja Majava, the proband exhibited apparent ocular and orofacial abnormalities with mild hearing loss, but abnormal skeletal system was not observed. In contrast, the mother of the proband exhibited isolated high myopia. Similarly, for our patient, myopia was the main presentation, but hearing abnormality was not observed. A similar situation in a heterozygous splice site variant (c.1845+5G>C and c.1845+5G>A) has also been observed (Brizola et al., 2020). Different variant sites or different base variants at the same variant site can produce different phenotypes. On the one hand, this may be because of the clinical heterogeneity among individuals; on the other hand, it may also be caused by the young age of the proband, who has not yet developed obvious symptoms. The proband has a mild phenotype at present, but the disease may progress in the future. Thus, we recommend that the proband needs regular hearing testing, with follow-up visits at ophthalmology and orthopedics departments. Although it is a *de novo* variant, prenatal diagnosis can be offered for parents of the proband in future pregnancies to prevent the recurrence of the same variant in other offspring owing to the presence of germline mosaicism.

## 5 Conclusion

We detected and identified a new pathogenic splicing variant, (NM_080629.3: c.4069-1G>T), in the *COL11A1* gene associated with STL. Our findings expand the variant spectrum of this gene and aid in the precise genetic diagnosis of STL. Gene tests on suspected cases may provide a firm diagnosis of STL. It could help in risk assessment, prophylaxis, and treatment of long-term complications of this disorder.

## Data Availability

The data presented in this article are not publicly available to assure patient confidentiality and participant privacy. Requests to access the datasets should be directed to the corresponding authors.

## Abbreviations

STL, Stickler syndrome; NGS, next-generation sequencing; WES, Whole-exome sequencing; BCVA, best-corrected visual acuity; ACMG, American College of Medical Genetics and Genomics; WT, wild type; MT, mutant type; RE, right eye; LE, left eye; 3D, Three-dimensional.
